# Bayesian spatial analysis of factors influencing neonatal mortality and its geographic variation in Ethiopia

**DOI:** 10.1371/journal.pone.0270879

**Published:** 2022-07-01

**Authors:** Getiye Dejenu Kibret, Daniel Demant, Andrew Hayen

**Affiliations:** 1 Department of Public Health, College of Health Sciences, Debre Markos University, Debre Markos, Ethiopia; 2 School of Public Health, Faculty of Health, University of Technology Sydney, Ultimo, NSW, Australia; 3 School of Public Health and Social Work, Faculty of Health, Queensland University of Technology, Brisbane, QLD, Australia; Universidade Federal de Minas Gerais, BRAZIL

## Abstract

**Background:**

Ethiopia is a Sub-Saharan country with very high neonatal mortality rates, varying across its regions. The rate of neonatal mortality reduction in Ethiopia is slow, and Ethiopia may not meet the third United Nations sustainable development target by 2030. This study aimed to investigate the spatial variations and contributing factors for neonatal mortality rates in Ethiopia.

**Methods:**

We analysed data from the 2016 Ethiopian Demographic and Health Survey (EDHS), which used a two-stage cluster sampling technique with a census enumeration area as primary and households as secondary sampling units. A Bayesian spatial logistic regression model using the Stochastic Partial Differential Equation (SPDE) method was fitted accounting for socio-economic, health service-related and geographic factors.

**Results:**

Higher neonatal mortality rates were observed in eastern, northeastern and southeastern Ethiopia, and the Somali region had higher risks of neonatal mortality. Neonates from frequently drought-affected areas had a higher mortality risk than less drought-affected areas. Application of traditional substances on the cord increased the risk of neonatal mortality (Adjusted Odds Ratio (AOR) = 2.07, 95% Credible Interval (CrI): 1.12 to 4.30) and getting health facility delivery services had a lower odds of neonatal mortality (AOR = 0.60, 95% CrI: 0.37, 0.98).

**Conclusions:**

Residing in drought-affected areas, applying traditional substances on the umbilical cord and not delivering at health facilities were associated with a higher risk of neonatal mortality. Policy-makers and resource administrators at different administrative levels could leverage the findings to prioritise and target areas identified with higher neonatal mortality rates.

## Introduction

The neonatal period is critical for newborn survival. The first seven days from birth constitute the early neonatal period, and the time from the seventh to the 28^th^ day is the late neonatal period [1].

In 2018, 2.5 million children died during their first month of life [2]. About 99% of these deaths were in low-and middle-income countries (LMIC) [3, 4], with Sub-Saharan Africa and South Asia accounting for 79% of all neonatal deaths [5]. Children born in Sub-Saharan Africa have ten times the risk of dying in their first month of life than children born in high-income countries [5]. Between 2017 and 2030, over 30 million newborn deaths are predicted globally, with more than half of these occurring in Sub-Saharan Africa [6].

The third Sustainable Development Goal (SDG) target is to reduce the neonatal mortality rate (NMR) below 12 deaths per 1000 live births by the end of 2030 [7]. In order to achieve this target, a significant acceleration of neonatal mortality reduction is required. In the 50 countries with the highest neonatal mortality rates, the annual rate of neonatal mortality reduction needs to be doubled between 2015 and 2030 to achieve the SDG target [8].

The Sub-Saharan Africa region has made the least progress in reducing neonatal deaths in the last three decades [3, 9]. In almost a third of the region’s countries, annual neonatal mortality rates were more than 30 deaths per 1000 live births in 2017, and two-thirds of countries that are at risk of missing the SDG neonatal mortality target are in sub-Saharan Africa [9].

Like most Sub-Saharan countries, Ethiopia has a high neonatal mortality rate [10–13]. In 2012, Ethiopia had the sixth-highest neonatal mortality rate globally, with an estimated death of 87,800 neonates or 31 deaths per 1000 live births [14, 15]. Ethiopia achieved the fourth millennium development target of reducing child mortality by two thirds, three years ahead of the 2015 deadline [16, 17]. The infant mortality rate also showed a promising decline, falling from 119 to 37 per 1000 live births between 1990 and 2019 [18]. However, the decline in neonatal mortality rate has been slow compared to the under-five and infant mortality reduction rates [19]. The United Nations (UN) Inter-agency Group for Child Mortality report indicated that the proportion of neonatal mortality in infant mortality increased from 51% in 1990 to 77% in 2019 in Ethiopia [18, 20]. Late neonatal and postneonatal mortality rates have also shown better reductions than early neonatal and overall neonatal mortality rates. The postneonatal mortality rate decreased by 60.4% from 2000 to 2016 [21, 22], and the late neonatal mortality rate decreased by 45% between 1995–2000 and 2006–2011. By contrast, early neonatal mortality decreased by 15% between 1995–2000 and 2006–2011 [23].

The current neonatal mortality reduction trends indicated that it would be difficult for Ethiopia to achieve the third SDG target of reducing neonatal mortality rates to below 12 deaths per 1000 live births by 2030 [24, 25]. In less developed countries, the lack of a well-organised Vital Registration (VR) system, as well as under-reporting of economic, epidemiological, and social data, make accurate statistical estimations difficult [26, 27]. The problem could be even worse in Ethiopia, where VR systems are still in their infancy, and the SDG objective on neonatal mortality reduction could be even more out of reach.

Evidence from studies on neonatal survival in Ethiopia report inconsistent neonatal mortality rates. A meta-analysis that pooled prevalence studied showed an overall neonatal mortality rate of 163.5 per 1000 live births [10]. A finding from another systematic review and meta-analysis of 11 studies conducted after 2012 in Ethiopia reported a neonatal mortality rate of 67.8 per 1000 live births [11], but with considerable heterogeneity. The variation in these findings might be due to the small sample size in the individual studies that addressed small areas of the country. In order to make noticeable progress in neonatal survival, policy-makers and resource administrators at all levels need to develop evidence-based strategies prioritising areas identified with high neonatal mortality rates [28].

**Fig 1:**
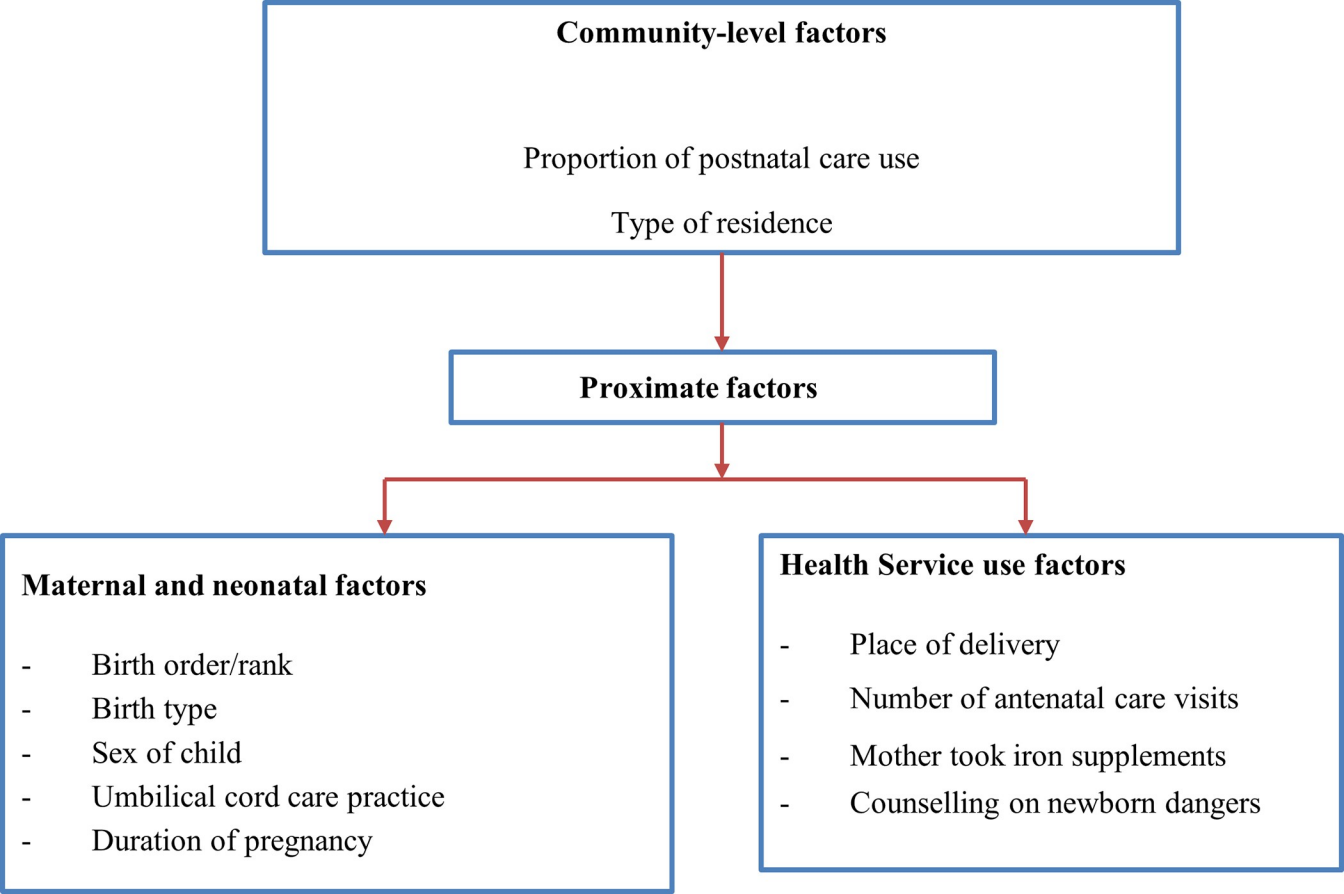
Conceptual framework of factors contributing to neonatal mortality, adapted from the Mosley and Chen framework for child survival [35].

A range of factors are associated with neonatal mortality, including community-level, socio-economic and proximal factors [28–34] (see Fig 1). The conceptual framework in Fig 1 incorporates socio-economic, proximal and geospatial factors that contribute to neonatal mortality. Proximal factors are factors related to health services use and neonatal and maternal characteristics. Findings from this study could inform policy-makers and local administrators to help better service administration and interventions where the problems are salient.

## Methods

### Study setting

Ethiopia is the second-most populous country in Africa, with a population of more than 112 million and a growth rate of 2.6% in 2019 [36]. The majority (80%) of the people reside in rural areas, with agriculture being the primary income source [37].

### Data source

The study is based on secondary data analysis of the 2016 Ethiopian Demographic and Health Survey (EDHS) [22]. The EDHS is a cross-sectional survey with a nationally representative sample. The datasets contain socio-economic, neonatal, maternal, geospatial and health service use related variables. The demographic, Global Positioning System (GPS) coordinates and geospatial data were combined using the cluster (enumeration area) code. Permission was granted to access the dataset through the Demographic and Health Surveys Program [38]. The detailed descriptions of the DHS design and sampling procedures can be found elsewhere [39].

### Study population and sampling

The target population of the study were newborns from birth to the 28^th^ day from birth in Ethiopia. The samples were selected using a two-stage cluster sampling technique using a census enumeration area (EA) as the primary sampling unit and households as the secondary sampling unit. An EA or cluster is a geographic area covering, on average, 181 households. In the 2016 EDHS survey, a total of 645 clusters were sampled. For the survey to be cost-effective and produce representative data at a national and sub-national level, the DHS applies an oversampling in regions with a small population and under-sampling in regions with a larger population. Therefore, DHS applies sampling weight to restore the representativeness of the samples and correct the deliberate under-sampling and over-sampling [40]. In the computation of means, totals, and percentages, we applied sample weighting based on the DHS recommendations [41].

The most recent births of mothers were included in the study to identify the factors associated with neonatal mortality because most of the health services use related variables such as antenatal care, place of delivery, and postnatal care were recorded for only the most recent live births.

**Fig 2:**
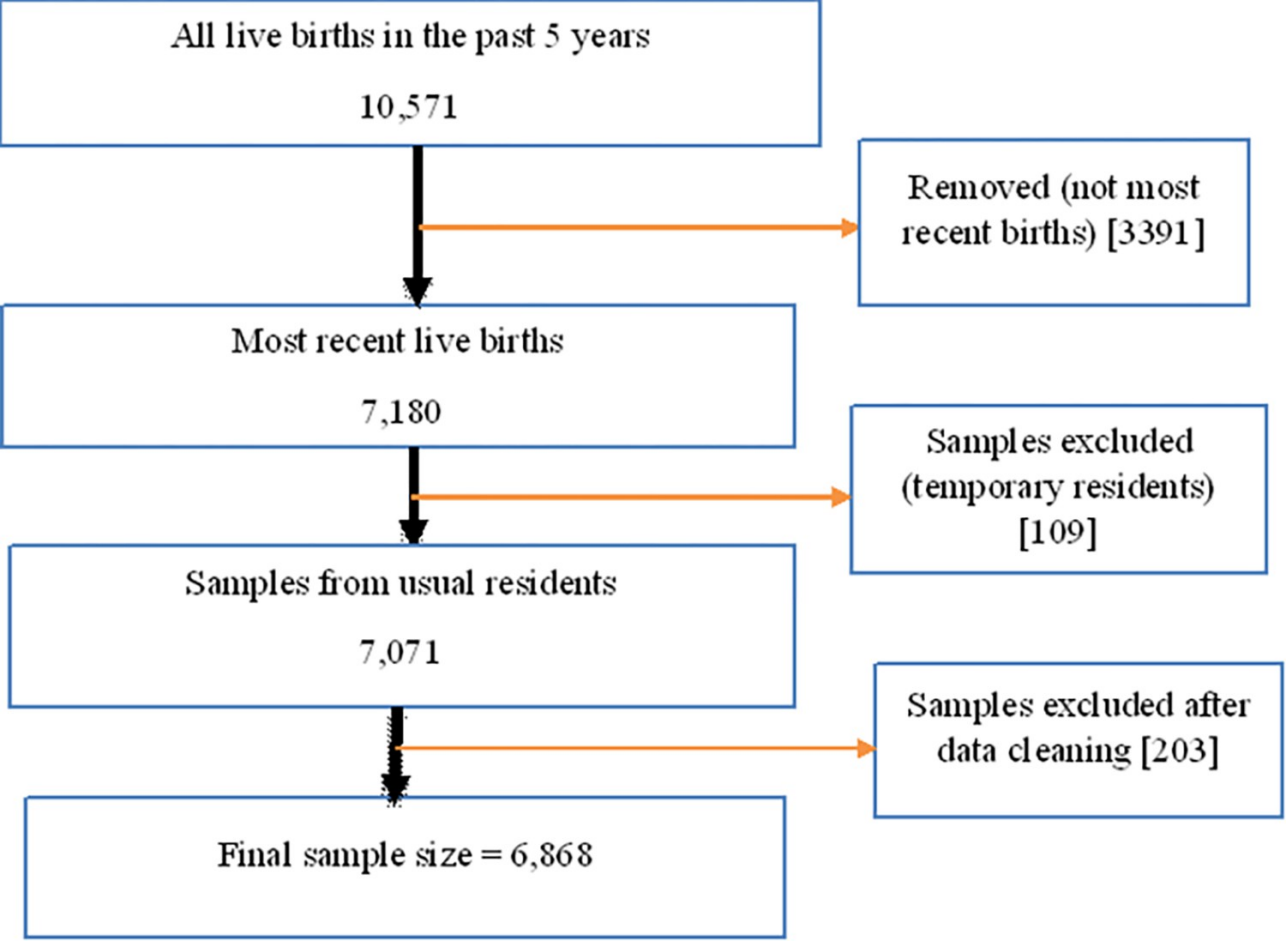
Flow chart of sample size determination for neonatal mortality in Ethiopia, 2016 EDHS.

The sampled total number of live births in the past five years of 2016 EDHS was 10,571, and health service use related data were collected from 7,180 most recent live births, of which 7,071 samples were collected from permanently residing respondents. One hundred nine survey participants were visitors and not included in the analysis. After data cleaning, the final analysis included 6,868 samples (see Fig 2).

### Study variables

We adopted the Mosley and Chen conceptual framework for child survival [35] to organise variables (see Fig 1). The primary outcome of interest was neonatal mortality, defined as death within 28 days of birth. The explanatory variables at the individual level include multiple births, birth order, child sex, umbilical cord care practice, antenatal care use, place of delivery, duration of pregnancy, counselling about neonatal danger signs and living situation of the mother. Factors such as residing in urban or rural areas, the proportion of postnatal care use per cluster and episodes of drought were considered at the community level (see S1 Table for definitions of variables).

## Data analysis

### Descriptive analysis

Percentages of socio-economic, maternal, neonatal, and health service-related variables and neonatal mortality were computed using a frequentist statistical approach. Frequencies of neonatal mortality across each categorical variable were calculated to investigate the sample distribution of the outcome variable. In the computation of means, totals, and percentages, we applied sample weighting based on the DHS recommendations [41].

### Analytical framework

A Bayesian spatial logistic regression model was fitted using the Stochastic Partial Differential Equation (SPDE) method. We applied the SPDE model in the Integrated Nested Laplace Approximation (R-INLA) package to estimate the determinants of neonatal mortality risks. The SPDE approach, along with INLA algorism, is a flexible and efficient method to analyse data collected over space with the desire to elicit an underlying pattern [42]. A regular grid of 66,778 pixels with a spatial resolution of 5 km^2^ was generated to predict neonatal mortality risks at unsampled locations and create a risk map. The Bayesian kriging method [43] was used to perform the predictions to the unsampled locations.

The Integrated Nested Laplace Approximation (INLA) algorithm in the R-INLA package [44] is a deterministic approach to approximate Bayesian inference for latent Gaussian models (LGMs), including the Bayesian generalised linear mixed (GLMM) models [45]. It is both faster and more accurate than Markov chain Monte Carlo (MCMC) alternatives for LGMs and can be used for quick and reliable Bayesian inference in practical applications [44].

The Empirical Bayesian Lasso (EBlasso) [46] logistic regression method was used for variable selection. Empirical Bayesian Lasso is a generalised linear regression method for variable selections and effect estimations. The package for the EBlasso algorithm outperforms other popular methods such as lasso and elastic net methods in terms of power of detection and false discovery rate [46, 47]. In the model building process, we set health services use variables such as antenatal care, facility delivery and postnatal care as a mandate to keep in the model and let the other variables be included in the model based on statistical variable selection criteria. Twenty-seven variables were identified from literature, and 13 variables were screened using the Empirical Bayesian Lasso algorithm and considered in the final model.

Separate models with spatial correlation (SPDE) and non-spatial (basic Bayesian logistic regression) were fitted, and the model that accounted for spatial correlation was the best fit model for the data. We compared the two models using the Watanabe-Akaike Information Criterion (WAIC) and Deviance Information Criterion (DIC).

Model fitness was assessed for the final Bayesian spatial model using the leave-one-out predictive measures, specifically the conditional predictive ordinates (CPO) [48] and the probability-integral transform (PIT) [49] statistics. The implicit assumptions in the INLA program were checked using the within-sample predictive accuracy measures (see S3 File). Analyses were carried out in R version 4.0.3 statistical software [50].

### Ethical considerations

Ethics approval was granted from the University of Technology Sydney Human Research Ethics Committee (HREC), which approved negligible risk to study participants (Approval Number: ETH19-4488). We also obtained approval to access the Demographic and Health Surveys dataset through the DHS Program. Informed written or verbal consent was not applicable since the study was based on data accessed from an online database.

## Results

### Socio-economic characteristics of study participants

A total of 6868 study samples were considered for analysis. The majority (87.4%) of the study participants were rural residents. Approximately two-thirds (63.5%) of the mothers did not have formal education, and 6,961 (96.3%) of the participants lived in households that used solid fuels such as wood, dung and charcoal for cooking. Less than a third (31.6%) of neonates are borne at health facilities (see Table 1).

**Table 1:** Socio-economic characteristics of study participants, EDHS 2016.

Variable		Sample Frequency	Proportion (weighted)	Number of neonatal deaths (n = 146)
Educational status of the mother	No education	4,182	63.5	98
	Primary education	1,840	28.2	36
	Secondary and above	846	8.4	12
Living situation of the mother	Living with partner	6,368	93.7	132
	Not living with a partner	500	6.3	14
Cooking energy used	Solid fuel	6,428	96.3	142
	Clean fuel	440	3.7	4
Household wealth tertiles	Highest	2,440	35.3	39
	Middle	973	20.9	20
	Lowest	3,455	43.7	87
Place of residence	Urban	1,440	12.6	17
	Rural	5,428	87.4	129
Region	Tigray	726	7.0	14
	Afar	626	1.0	12
	Amhara	708	20.9	14
	Oromia	997	41.8	25
	Somali	794	3.7	31
	Benishangul	546	1.1	9
	SNNPR	848	21.1	10
	Gambela	509	0.3	11
	Harari	400	0.2	10
	Addis Ababa	355	2.6	3
	Dire Dawa	359	0.4	7

The median age of mothers was 28.0 years, with an interquartile range of 25 to 34 years. More than half (58.2%) of the study participants did not take iron supplements during their recent pregnancy, and the majority (89.0%) did not receive counselling services on newborn danger signs within the first two days of the postpartum period. Traditional substances such as dung, oil, and different ointments were applied as cord care for most newborns (80.4%) (see Table 2).

**Table 2:** Maternal, neonatal and health service-related (proximate factor) characteristics.

Variable		Sample Frequency	Proportion (weighted)	Number of neonatal deaths (n = 146)
Sex of child	Male	3,561	52.2	97
	Female	3,307	47.8	49
Birth type	Singleton	6,761	98.4	128
	Twin	107	1.6	18
Umbilical cord care	Nothing applied	1,015	14.4	10
	Substances applied	5,473	80.4	124
	Don’t know	380	5.2	12
Mother took iron supplements during pregnancy	No	3,750	58.2	98
	Yes	3,100	41.7	48
Health provider counseled on newborn dangers	No	6,000	89.0	136
	Yes	868	11.0	10
Size of the neonate at birth	Average	2,886	27.2	46
	Large	2,097	32.0	57
	Small	1,885	40.8	43
Number of antenatal care visits	4 or more	2,471	31.8	37
	1 to 3	1,996	30.6	30
	No antenatal care	2,401	37.6	79
Place of delivery	Health facility	2,592	31.6	49
	Home	4,276	68.4	97
Tetanus protection at birth	Protected	2,161	31.1	39
	1–2 injections	2,249	33.8	34
	No injection	2,458	35.2	73
Duration of pregnancy	Preterm	117	1.3	20
	Term	6,751	98.7	126
Preceding birth interval	More than two years	4,202	64.9	84
	Two years or less	1,283	16.8	35
	First birth	1,383	18.3	27

### Neonatal mortality rate

**Fig 3:**
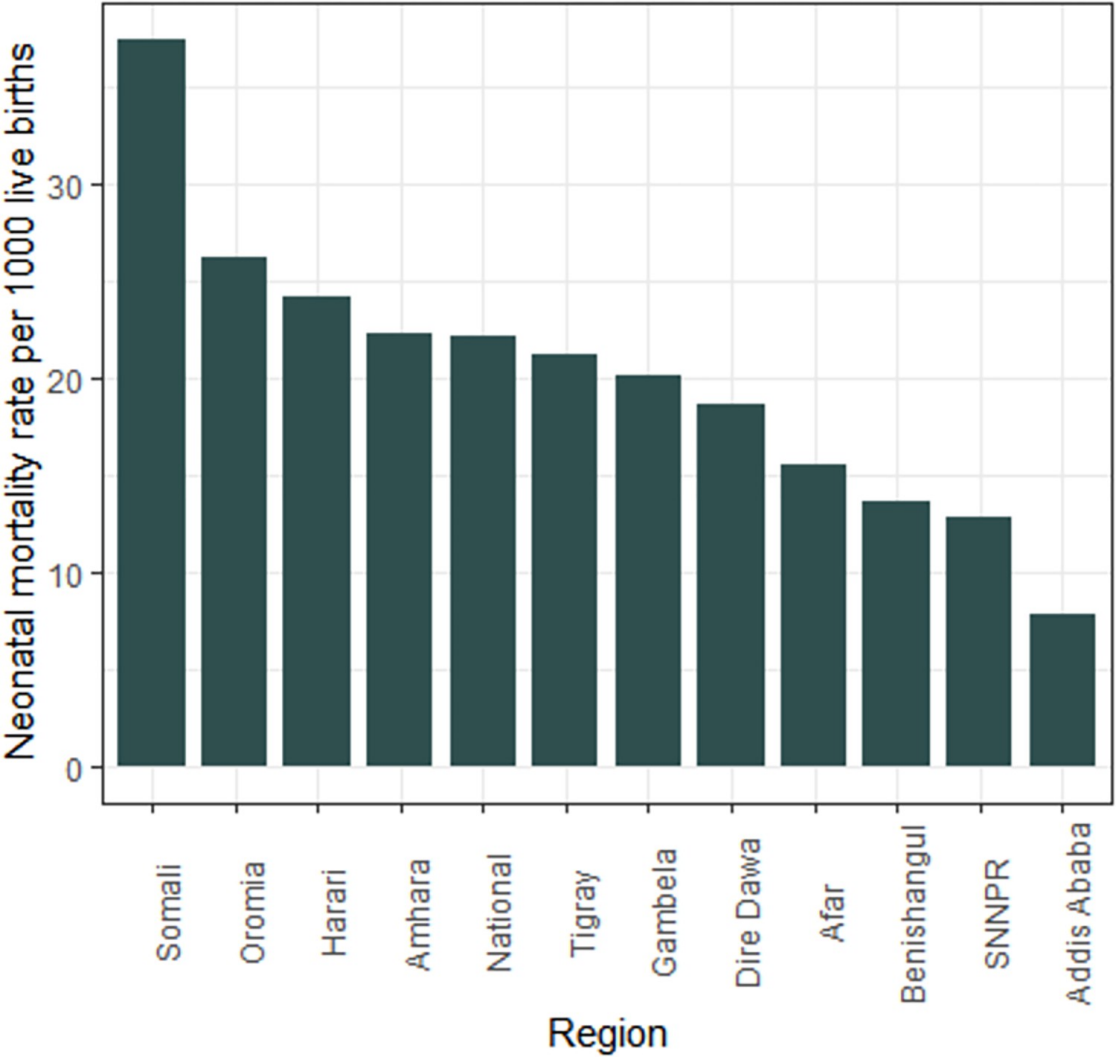
Regional neonatal mortality rates per 1000 live births among most recent births in the past five years before the survey, EDHS 2016.

One hundred forty-six neonatal deaths were observed, with 22.2 deaths per 1000 live births (95% CI: 17.1 to 27.9) for the most recent live birth in the past five years of the survey. The highest mortality rate was in the Somali region, with 36.8 deaths per 1000 live births and the lowest mortality rate was in Addis Ababa with 8.0 deaths per 1000 live births (Fig 3).

### Factors associated with neonatal mortality

Effects of covariates were estimated after controlling for confounders and spatial correlations. The spatial random effects (SPDE-INLA) model explains a mean-variance of 0.16 over the basic Bayesian logistic regression model. We found that neonates from frequently drought-affected areas had a higher mortality risk than less frequently affected areas (AOR = 1.58,95% CI: 1.10 to 2.73). Application of traditional substances on the cord increased the risk of neonatal mortality (AOR = 2.07, 95% CrI: 1.12 to 4.30) compared to those who did not apply traditional substances. Neonates born at health facilities had a lower rate of mortality than those born at home (ARO = 0.60, 95% CrI: 0.37 to 0.98). In our study, less than a third of neonates were born in health facilities.

Twin pregnancies had a higher risk of neonatal mortality (AOR = 8.40, 95% CrI: 4.45, 14.90) than singletons, and female neonates were 73% less likely to die than males (AOR = 0.27, 95% CrI: 0.19 to 0.38). Term births had a lower risk of mortality compared to preterm births (AOR = 0.08, 95% CrI: 0.05 to 0.15). Newborns born to mothers residing in rural areas had a higher risk of neonatal mortality than those who lived in urban areas (AOR = 2.38, 95% CrI: 1.31 to 4.57) (see Table 3).

**Table 3:** Factors influencing neonatal mortality in Ethiopia, EDHS 2016.

Variable	Category	Adjusted Odds Ratio (95% Credible Interval)
Sex of child	Male	1
	Female	**0.27 (0.19, 0.38)**
Birth type	Singleton	1
	Twin	**8.40 (4.45 14.90)**
Birth order		1.06 (0.99, 1.13)
Umbilical cord care	Nothing applied	1
	Substances applied	**2.07 (1.12, 4.30)**
	Do not know	4.58 (1.48, 8.89)
Mother took iron supplements during pregnancy	No	1
	Yes	0.82 (0.53, 1.27)
Number of antenatal care visits	4 or more	1
	1 to 3	0.91 (0.54, 1.52)
	No antenatal care	1.67 (0.97, 2.92)
Duration of pregnancy	Preterm	1
	Term	**0.08 (0.05, 0.15)**
Place of delivery	At home	**1**
	Health facility	**0.60 (0.37, 0.98)**
Place of residence	Urban	1
	Rural	**2.38 (1.31, 4.57)**
Living situation of the mother	Living with partner	1
	Not living with a partner	1.52 (0.79, 2.65)
Number of antenatal care visits	4 or more	1
	1 to 3	0.91 (0.54, 1.52)
	No antenatal care	1.67 (0.97, 2.92)
Proportion of postnatal care use per cluster		0.33 (0.05, 1.87)
Episodes of drought in 21 years period	Five or fewer episodes	1
	More than five episodes	**1.58 (1.10, 2.73)**

**Fig 4:**
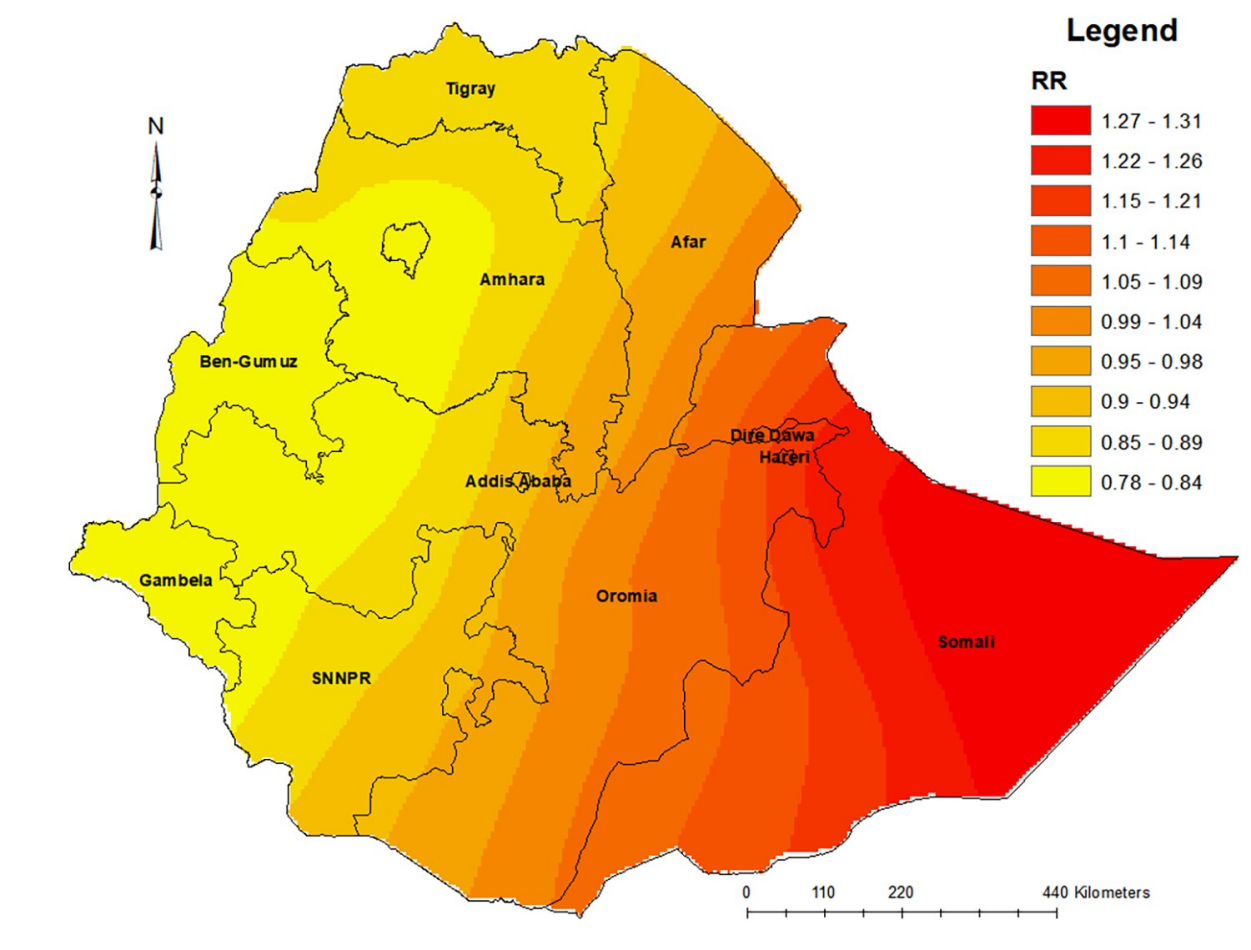
Posterior distributions of neonatal mortality risks in Ethiopia after controlling for socio-economic, neonatal, maternal, health service use and geographic covariates, EDHS 2016, (source: Natural Earth [51]).

There were also variations in risks of neonatal mortality among the regions. After controlling for the socio-economic, neonatal, maternal, health service use and geographic factors, the spatial distribution showed higher risks of neonatal mortality in the eastern, northeastern and southeastern parts of Ethiopia. The risks of neonatal mortality tail off while moving from Eastern to the Western parts of the country. The Somali region had a higher risk of neonatal mortality (see Fig 4).

## Discussion

We found that most parts of the Somali region had higher risks of neonatal mortality, which is in line with the higher incidence of neonatal mortality in that state identified in other reports [13]. This might be related to the limited infrastructure and health service coverage in the state. The 2014 service provision and availability survey report indicated that the Somali regional state was one of the worst-performing, with health facilities infrastructure coverage [52]. Most of the Somali regional areas are also dry [53], which has been identified as a contributing factor to neonatal mortality in this study.

The frequency of drought was associated with neonatal mortality. Neonates from frequently drought-affected areas had a higher risk of mortality. This may have resulted from shortages of precipitation that led to recurrent and substantial shortfalls in agricultural production and claimed tens of thousands of human and animal lives [54]. This is particularly meaningful as Ethiopia’s economy is predominantly reliant on rain-dependent farming and livestock. Over 15 million agro-pastoralists in Ethiopia are herding livestock in drought-prone areas. Most of the drought and food crisis events have been geographically concentrated in two broad zones, the country’s eastern and northern parts [55]. The country’s dependency on agriculture, combined with repeated drought-hit and ongoing political fragility, negatively impacts its agro-economic and health care systems [56].

Neonates born at health facilities had a lower mortality rate than those born at home. In our study, the proportion of neonates born in health facilities accounted for less than a third of all births. Consistent with our findings, the current body of literature has shown a link between neonatal death and place of delivery. A meta-analysis of studies from developing countries showed that mothers who gave birth in health facilities had a 52% lower risk of neonatal mortality than mothers who gave birth at home [57]. Another meta-analysis found that giving birth in a health facility reduced the risk of neonatal mortality by 29% [58].

The application of traditional substances on the umbilical cord was found to increase neonatal mortality. Some of the traditional substances commonly applied in the study area were dung, oil, ointment, and butter, which can increase bacterial colonisation of the umbilical wound [59, 60]. Neonatal sepsis usually stems from local umbilical cord infections and later becomes systemic, one of the most typical causes of neonatal deaths [61].

The current study showed that neonates born to mothers residing in rural areas were at higher risk of neonatal mortality than those living in urban areas. This finding is in line with a meta-analysis finding in Ethiopia [10], a study in Nigeria [34] and another study from Gansu province China [62]. This might be due to rural areas’ restricted access to obstetric and neonatal care services, such as low institutional and caesarian delivery rates [63], insufficient sanitation and water supply, and a lower proportion of neonatal care education and awareness [64].

The risk of neonatal mortality was higher among twin pregnancies compared to singletons. Multiple pregnancies carry an extra risk for both the mother and babies and increase the risk of pregnancy-induced hypertension [65, 66], bleeding during pregnancy [65], congenital abnormalities, and fetal growth retardation, especially in the third trimester due to the increased fetal demands [66]. Multiple pregnancies can also lead to preterm labour, thereby low birth weight, which is the main contributing factor for neonatal mortality [67, 68]. Generally, multiple pregnancies are long known for owing a higher risk of pregnancy and delivery complications and a higher probability of birth defects and infections [69]. Moreover, neonatal survival decreases further when multiple pregnancies are combined with a lack of access to basic and advanced health care services, which is the case in low-income countries like Ethiopia [70].

The study showed that male neonates had a higher risk of mortality during the first 28 days of life than female neonates. This finding agrees with the current body of literature that reports male neonates to have a higher risk of death than female neonates. Evidence showed that male sex neonates are disadvantageous to survive in cases of low birth weight, premature birth, and multiple births than their equivalent female neonates [71–78].

Preterm births were highly associated with neonatal mortality in this study. Evidence showed that preterm birth occurs in 5 to 10% of all pregnancies and accounts for 75% of neonatal morbidity and 70% of neonatal deaths [79, 80], as premature newborns are highly vulnerable to infections [81]. Furthermore, infants born preterm are more likely to have an increased risk of neurological impairment and pulmonary disorders [80]. On the other hand, the provision of intensive care for preterm newborns is an enormous burden [79] on the healthcare system.

### Limitations of the study

As the current study was based on secondary data analysis, there is the possibility of reporting and recall bias for retrospective data relying on memory of a past event. Health service use related factors were collected only for the most recent births, limiting the scope of analysis. Moreover, there were omissions of values for some variables limiting the exhaustive consideration of the determinants. Further, the place of residence at the time of giving birth may have been different to when the participants were surveyed.

## Conclusions

Areas in the eastern, northeastern and southeastern parts of Ethiopia were identified with higher neonatal mortality rates. The Somali region and neighbouring areas had higher mortality rates. Neonates from frequently drought-affected areas, giving birth at home and applying traditional substances to the umbilical were associated with higher neonatal mortality rates.

Policy-makers and resource administrators at different levels could leverage the findings to prioritise and target areas identified with higher neonatal mortality rates. Health professionals should promote facility delivery services and safe umbilical cord care in the early days of birth. Policy intervention should be in place to integrate umbilical cord care into the health extension program packages. Improving access to effective obstetric and neonatal care and strengthening health services for neonates with preterm and twin births can improve neonatal survival.

## Supporting information

S1 Table Descriptions of study variables for factors associated with neonatal mortality. (DOCX)

S1 Fig Hotspot clusters of neonatal mortality in Ethiopia, EDHS 2016. (TIF)

S2 Fig Sample clusters for Ethiopian Demographic and Health Surveys, EDHS 2016. (TIF)

S1 File Analysis R script for Bayesian special analysis. (PDF)

S2 File Model formulation for Bayesian spatial logistic regression. (DOCX)

S3 File Model validation and comparison. (DOCX)
