# Supplementary material for: Bayesian spatial analysis of factors influencing neonatal mortality and its geographic variation in Ethiopia
Source: PLoS One. 2022 Jul 1;17(7):e0270879. doi: 10.1371/journal.pone.0270879 (PMC9249191; doi:10.1371/journal.pone.0270879)
Supplement: S1 Table — (DOCX) [file pone.0270879.s001.docx]

**Descriptions of study variables for factors associated with neonatal mortality in Ethiopia, EDHS 2016**

| **Variable** | **Value levels** | **Descriptions** |
| --- | --- | --- |
| Neonatal survival status | 1. Survived 2. Dead | Outcome variable defined either death within 28 days of birth or survived beyond |
| **Community-level factors** | | |
| Cluster |  | A cluster is a geographic area covering, on average, 181 households. Cluster is, also known as census enumeration area, is used as a sampling frame |
| Place of residence | 1. Urban 2. Rural |  |
| Proportion of postnatal care use |  | Postnatal care use with two days of birth |
| Episodes of drought | 1. Five or fewer episodes 2. More than five episodes | The average number of drought episodes (categorised between 1 (low) and 10 (high)) for the areas within the 2 km (urban) or 10 km (rural) buffer surrounding the DHS survey cluster location-based on, utilising data from 1980 through 2000 for 21 years |
| Travel time to towns with 50,000 or more people settlement | 1. Two or fewer hours 2. More than two hours | The average time (minutes) required to reach a settlement of 50,000 or more people from the  the area within the 2 km (urban) or 10 km (rural) buffer surrounding the DHS survey cluster location, based on the year 2015 infrastructure data |
| **Socio-economic variables** | | |
| Living situation of the mother | 1. Living with a partner 2. Not living with a partner | Women who are divorced/separated/widowed are considered not living with a partner |
| Educational status of the mother | 1. No education 2. Primary education 3. Secondary and above | The interval between the current birth and preceding births |
| Household wealth index | 1. poor 2. middle 3. rich | Wealth index: is a composite measure of a household cumulative living standard, calculated using easy-to-collect data on a household’s ownership of selected assets, such as televisions and bicycles; materials used for housing construction etc. |
| Cooking energy used | 1. Solid fuel 2. Clean fuel | Solid fuels include coal/lignite, charcoal, wood, straw/shrub/grass, agricultural crops, and animal dung  Clean fuels include electricity, liquefied petroleum gas (LPG), natural gas, and biogas |
| **Proximate determinants** | | |
| **Maternal and neonatal factors** | |  |
| Birth order/rank |  |  |
| Birth type | 1. Singleton 2. Twin |  |
| Sex of child | 1. Male 2. Female |  |
| Umbilical cord care practice | 1. Nothing applied 2. Traditional substances 3. Don’t know | Traditional substances applied on a cord: dung, oil, ointment |
| Duration of pregnancy | 1. Preterm 2. Term | Preterm: 8 months and below  Term: 9 months and above |
| Size of the neonate at birth |  | mother’s estimate of the baby’s size at birth |
| Preceding birth interval | 1. Greater than 24 months 2. 24 months or less 3. First birth |  |
| **Health service use related factors** | | |
| Place of delivery | 1. Health facility 2. Home 3. Other | Health facility includes all private and public health facilities, including hospitals, health centres, clinics, health posts. |
| Number of antenatal care visits | 1. 4 or more 2. 1-3 3. No antenatal care | WHO recommended at least four antenatal care visits |
| Mother took iron supplements during pregnancy | 1. No 2. Yes | Given or bought iron tablet/syrup during pregnancy |
| Health provider counsel on newborn dangers in the first two days | 1. No 2. Yes 3. Do not know |  |
| Tetanus protection at birth | 1. Protected 2. Not protected | A birth is protected against neonatal tetanus if the mother has received any of the following:  - Two tetanus toxoid injections during that pregnancy or,  - Two or more injections, the last one within three years of the birth or,  - Three or more injections, the last one within five years of the birth or,  - Four or more injections, the last one within ten years of the birth or,  - Five or more injections at any time prior to the birth |
