# Supplementary figures and images for: Bayesian spatial analysis of factors influencing neonatal mortality and its geographic variation in Ethiopia
Source: PLoS One. 2022 Jul 1;17(7):e0270879. doi: 10.1371/journal.pone.0270879 (PMC9249191; doi:10.1371/journal.pone.0270879)

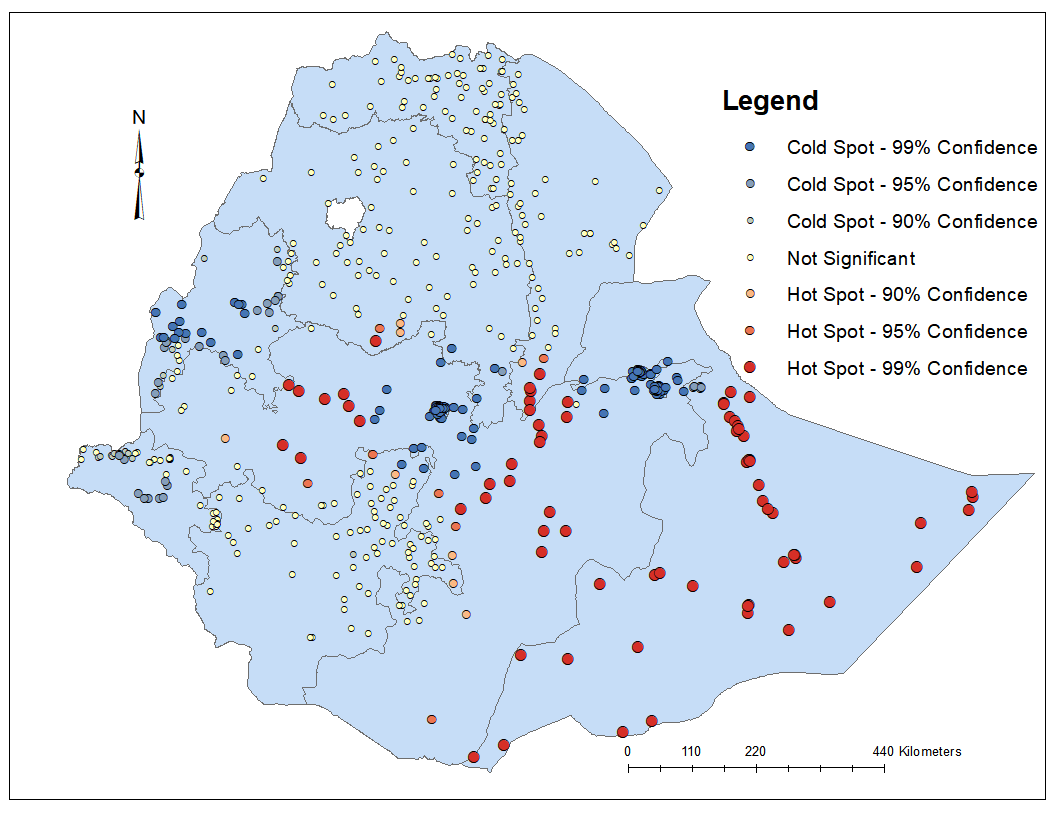

Supplement: S1 Fig — (TIF) [file pone.0270879.s002.tif]

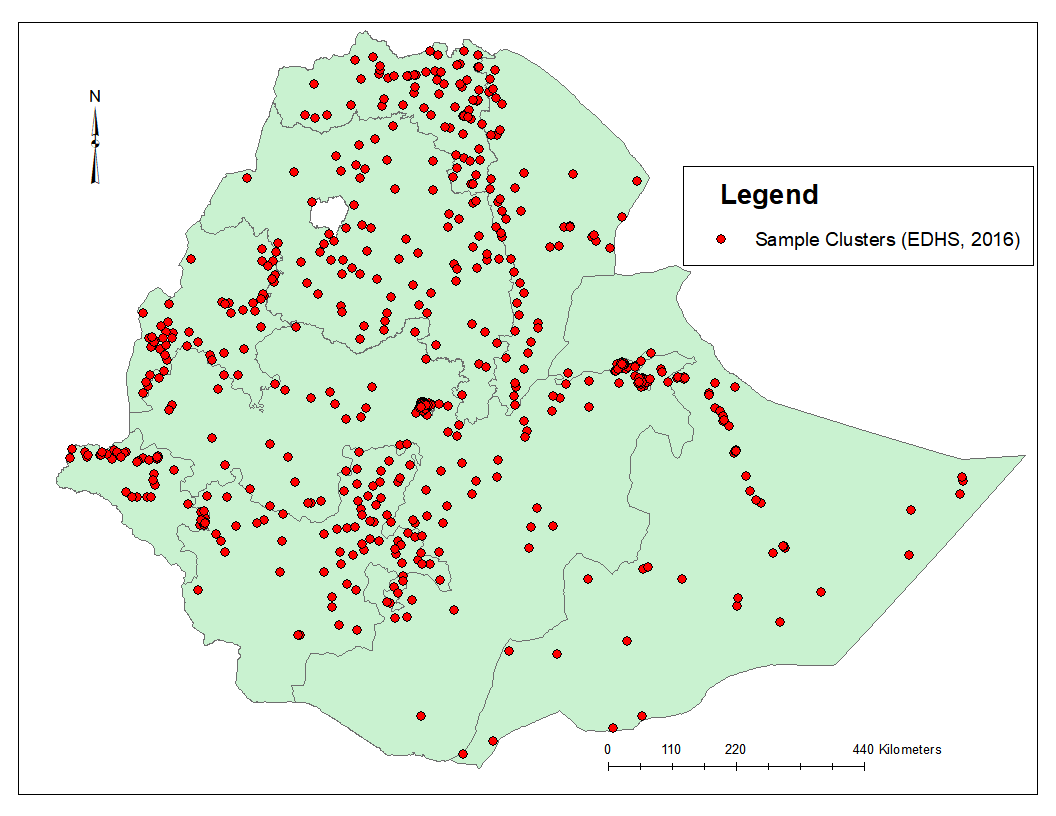

Supplement: S2 Fig — (TIF) [file pone.0270879.s003.tif]
