# Supplementary material for: Bayesian spatial analysis of factors influencing neonatal mortality and its geographic variation in Ethiopia
Source: PLoS One. 2022 Jul 1;17(7):e0270879. doi: 10.1371/journal.pone.0270879 (PMC9249191; doi:10.1371/journal.pone.0270879)
Supplement: S1 File — (PDF) [file pone.0270879.s004.pdf]

Bayesian spatioal analysis R script

Loading up the libraries and data we need

```
knitr::opts_chunk$set(echo = FALSE)

# loading  libraries
library(rgdal)
library(dplyr)
library(tidyverse)
library(sf)
library(INLA)
library(EBglmnet)
library(INLAutils)
library(here)



# loading data
df<- read.csv("C:/Users/dgeti/OneDrive - UTS/UTS/Output_1/review  1/demographic_covs.csv") #Socio-demographic dat
a
geo <- readOGR(dsn = "C:/Users/dgeti/OneDrive - UTS/UTS/Output_1/review  1/dataset", "2016",
               verbose = FALSE)
adm0 <- readOGR(dsn = "C:/Users/dgeti/OneDrive - UTS/UTS/Output_1/review  1/dataset",
                "Eth_Boundary", verbose = FALSE)
adm1<-readOGR(dsn = "C:/Users/dgeti/OneDrive - UTS/UTS/Output_1/review  1/dataset",
              "sdr_subnational_boundaries", verbose = FALSE)
geocov<- read.csv(file=here("geospatial_cov_imputed.csv"))
#ethio <- raster(file=here("etnachrpprecchng.tif"))
```

Convert the DHS shape file to sf file and merge to DHS dataset file

```
dat1<-st_as_sf(geo)  #dhs shp file to sf file
geo.df <- merge(dat1, df, by.x = "DHSCLUST", by.y = "V001")
geo_df_geocov <- merge(geo.df, geocov, by.x= "DHSCLUST", by.y="dhsclust")
dat2<-st_as_sf(geo_df_geocov) #convert to dataframe
```

Variable selection using Penalised Bayesian logistic regression model in "EBglmnet" package

```
da <- dat2 %>% st_drop_geometry()
y= da[,"death"]
xNames = c('V024','V025', 'V190','BORD' ,'B0', 'B4', 'M45','M78C',
           'education','S434A','cooking','size','delivery_place', 'anc_vist',
           'duration',  'c_tetanus_p', 'c_place_delivery_p','tetanus_prot',
           'c_pnc_p', 'mean_anc', 'drought_cat', 'global_hfo_cat',
            'V212', 'V467D', 'aridity_cat',  'media', 'marital')

x = as.matrix(da[,xNames])
out = cv.EBglmnet(x,y,family="binomial" ,prior= "lasso",
                  Epis = FALSE,group = FALSE, verbose = 0)
```

```
out$fit
```

```
##       locus1 locus2        beta posterior variance   t-value      p-value
##  [1,]      2      2  0.09909388       0.0125371093 0.8850099 3.761834e-01
##  [2,]      4      4  0.03883331       0.0006898136 1.4785584 1.393058e-01
##  [3,]      5      5  1.04843639       0.0222460471 7.0293580 2.282174e-12
##  [4,]      6      6 -0.40020194       0.0219898645 2.6987827 6.976978e-03
##  [5,]      7      7 -0.08973163       0.0132779703 0.7787171 4.361741e-01
##  [6,]      8      8 -0.38105358       0.0689027718 1.4516693 1.466408e-01
##  [7,]     10     10  0.08349315       0.0016373547 2.0633812 3.911522e-02
##  [8,]     14     14  0.25954092       0.0111394731 2.4590848 1.395451e-02
##  [9,]     15     15 -2.07077113       0.0810641023 7.2730707 3.916867e-13
## [10,]     17     17 -0.07095315       0.0189788001 0.5150357 6.065452e-01
## [11,]     19     19 -0.96537377       0.4386070044 1.4576650 1.449802e-01
## [12,]     21     21  0.18588096       0.0239189776 1.2018869 2.294502e-01
## [13,]     27     27  0.14061810       0.0376450560 0.7247484 4.686320e-01
```

Spatial Component

Extract covariates for organising the geospatial data

```
final_cov<- select(dat2, c(DHSCLUST, LATNUM,LONGNUM, V024,V025, V190, BORD,B0, B4, M45,M78C,
                          education,S434A,size,delivery_place,anc_vist,
  tetanus_prot,duration,marital,death,aridity_2015, c_tetanus_p, c_place_delivery_p,c_pnc_p,
  mean_anc,aridity_cat , drought_cat, global_hfo_cat,travelItime_cat,rainfall_2015))
```

Transforming coordinates

```
xy <- final_cov[,c(2,3)]
dat_coords <- st_as_sf(x = final_cov, #data with coordinates
                      coords = xy,
                      crs = "+proj=longlat +datum=WGS84 +ellps=WGS84 +towgs84=0,0,0")
# convert to sp object if needed
sps <- as(dat_coords, "Spatial")

spst <- spTransform(sps, CRS("+proj=longlat +datum=WGS84"))
```

Adding the longitude and latitude coordinates to the dataframe

```
dat_coords[, c("LONGNUM", "LATNUM")] <- coordinates(spst)
dat_coords[, c("x", "y")] <- coordinates(spst)
points.sf <- st_as_sf(spst)
bdry.sf <- st_as_sf(adm0)
```

Mesh Construction

Mesh/Triangulation is the first step to fit the spatial model. It is like choosing the integration points on a numeric integration algorithm- creating triangular nodes

```
# Scale the spatial coordinates to make mesh construction easier
coo <- cbind(dat_coords$LONGNUM, dat_coords$LATNUM)
adm0 <- spTransform(adm0, CRS("+proj=longlat +datum=WGS84"))
bdry <- inla.sp2segment(adm0)
```

```
## Warning in proj4string(sp): CRS object has comment, which is lost in output
```

```
bdry$loc <- inla.mesh.map(bdry$loc)

mesh <- inla.mesh.2d(loc = coo, boundary = bdry, max.edge=c(0.5, 1),
                     #min.angle=c(20, 20),
                     max.n=c(48000, 16000), ## Safeguard against large meshes.
                     max.n.strict=c(128000, 128000), ## Don't build a huge mesh!
                     cutoff=0.1, ## Filter away adjacent points.
                     offset=c(0.5, 1)) ## Offset for extra boundaries, if needed.
plot(mesh)
points(coo, col = "red")
```

Constrained refined Delaunay triangulation

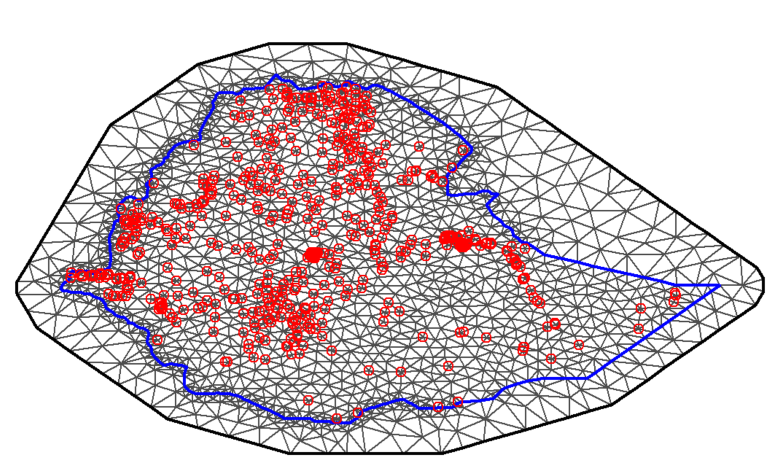

Projection matrix (A mtrix):

The projection matrix makes the link between the observed data and the spatial effect
estimated by the model
The A matrix maps the Gaussian Markov Random Field (GMRF) from the mesh nodes to the n observation locations

```
A<-inla.spde.make.A(mesh=mesh,loc=as.matrix(coo))
```

Building SPDE

Create the spatial structure (SPDE object) - defined on the mesh

```
spde <- inla.spde2.matern(mesh, alpha=2)
```

The SPDE object is approximate at the mesh node
Parameter alpha is related to the smoothness parameter of the process
We set the smoothness parameter equal to 1 and in the spatial case d=2 so alpha=1+2/2=2

Index set for the SPDE model

Then we generate the index set for the SPDE model, Where we specify the name of the effect and the number of vertices in the SPDE model

```
iset <- inla.spde.make.index(name = "spatial.field", spde$n.spde)
```

Create INLA stack

The INLA stack is way of creating a list of items required to build a model
The three main inla.stack() arguments are a vector list with the data,
a list of projector matrices (each related to one block effect, A) and the list of effects

```
stk <- inla.stack(data=list(y=dat_coords$death, n=dat_coords$B4), #the response

                  A=list(A,1),

                  effects = list(c(iset, list(Intercept = 1)),
                                 list(
                                   cluster=dat_coords$DHSCLUST,
                                   latitude=dat_coords$LATNUM,
                                   longitude=dat_coords$LONGNUM,
                                   residence=factor(dat_coords$V025),
                                   birth_order=dat_coords$BORD,
                                   twin=factor(dat_coords$B0),
                                   sex=factor(dat_coords$B4),
                                   iron=factor(dat_coords$M45),
                                   postnatal_c =dat_coords$c_pnc_p,
                                   umbilical_care=factor(dat_coords$S434A),
                                   anc=factor(dat_coords$anc_vist),
                                   duration=factor(dat_coords$duration),
                                   drought= factor(dat_coords$drought_cat),
                                   marital=factor(dat_coords$marital),
                                   counselling=factor(dat_coords$M78C),
                                   delivery_place=factor(dat_coords$delivery_place)
                                    )),

                  # we can tag a quick name to call easily
                  tag='est')
```

Fitting the Spatial model

```
formula1 <- y ~  birth_order + sex+ iron+
    postnatal_c + twin  + umbilical_care  +anc+ delivery_place +
  residence +  drought + duration+  marital+ counselling +
  f(cluster, model = "iid") + f(spatial.field, model=spde)

model1<-INLA::inla(formula1, #the formula
             data=inla.stack.data(stk,spde=spde),  #the data stack
             family= 'binomial',   #which family the data comes from
             Ntrials = n,      #this is specific to binomial as we need to tell it the
             #number of examined
             control.predictor=list(A=inla.stack.A(stk),compute=TRUE),  #compute gives the
             # marginals of the linear predictor
             control.compute = list(dic = TRUE, waic = TRUE, config = TRUE, cpo=TRUE),
             #model diagnostics and config = TRUE gives #the GMRF, cpo = TRUE provides the model fitness parametr
es.
             verbose = FALSE) #can include verbose=TRUE to see the log of the model runs


round(exp(model1$summary.fixed[-1, c(4,3,5)]),2)
```

```
##                 0.5quant 0.025quant 0.975quant
## birth_order         1.06       0.99       1.13
## sex2                0.27       0.19       0.38
## iron1               0.82       0.53       1.27
## postnatal_c         0.33       0.05       1.87
## twin2               8.40       4.45      14.90
## umbilical_care1     2.07       1.12       4.30
## umbilical_care8     3.58       1.48       8.89
## anc1                0.91       0.54       1.52
## anc2                1.67       0.97       2.92
## delivery_place1     0.60       0.37       0.98
## residence2          2.38       1.31       4.57
## drought1            1.58       1.10       2.73
## duration1           0.08       0.05       0.15
## marital1            1.52       0.79       2.65
## counselling1        0.53       0.23       1.04
```

Model posterior variance estimation-variance of the random effects

```
# Transformation of the marginal
marg.variance <- inla.tmarginal(function(x) 1/x,
model1$marginals.hyperpar$"Precision")

# Mean posterior of the variance
m <- inla.emarginal(function(x) x, marg.variance)
m
```

```
## [1] 0.1627217
```

Fit model without spatial correlation (Bayesian Logistic regresion)

```
formula2 <- death~ V025 +BORD +B0 +B4 +M45 + M78C+
  S434A+ anc_vist +duration +
  c_place_delivery_p +c_pnc_p +
  drought_cat + marital+ f(DHSCLUST, model = "iid")


model2 <-inla(formula2, family="binomial",
              data=final_cov, control.compute=list(dic=TRUE,waic=TRUE))
```

Compare models with and with out spatial component

```
c(model1$waic$waic, model2$waic$waic)
```

```
## [1] 1229.105 1234.968
```

RF projection on a grid

```
# Predict  grid
points.em <- mesh$loc

stepsize <- 5 * 1 / 111 # This is given in coordinates unit (in this case this is straightforward and correspond
 to 150m)
x.range <- diff(range(points.em[,1]))  # calculate the length of the x range
y.range <- diff(range(points.em[,2])) # calculate the length of the y range

nxy <- round(c(x.range, y.range)/stepsize)  # Calculate the number of cells in the x and y ranges

### Project the spatial field on the mesh vertices using the inla.mesh.projector() function
projgrid <- inla.mesh.projector(mesh,
                                xlim = range(points.em[,1]),
                                ylim = range(points.em[,2]),
                                dims = nxy)
##removing points outside the boundary
xy.in <- inout(projgrid$lattice$loc, bdry$loc)
table(xy.in)

#projection of posterior mean and posterior standard deviation
xmean <- inla.mesh.project(projgrid,model1$summary.random$spatial.field$mean)
xsd <- inla.mesh.project(projgrid, model1$summary.random$spatial.field$sd)
#points that fall outside the boundaries are set to have a missing values
xmean[!xy.in] <- NA
xsd[!xy.in] <- NA

###creating raster spatial objects for the mean, sd and standard deviation of the GRF.
#estimated mean
xmean2 <- t(xmean)
xmean3 <- xmean2[rev(1:length(xmean2[,1])),]
xmean_ras <- raster(exp(xmean3),#exponenting the logit to RR (OR)
                    xmn = range(projgrid$x)[1], xmx = range(projgrid$x)[2],
                    ymn = range(projgrid$y)[1], ymx = range(projgrid$y)[2],
                    crs = CRS("+proj=longlat +datum=WGS84"))

writeRaster(xmean_ras, filename = "xmean_ras.tif", overwrite=TRUE)
###standard deviation
xsd2 <- t(xsd)
xsd3 <- xsd2[rev(1:length(xsd2[,1])),]
xsd_ras <- raster(xsd3,
                  xmn = range(projgrid$x)[1], xmx =range(projgrid$x)[2],
                  ymn = range(projgrid$y)[1], ymx =range(projgrid$y)[2],
                  crs = CRS("+proj=longlat +datum=WGS84"))
### saving raster items stored and  manipulated outside R (eg  GIS) for visualisation
writeRaster(xsd_ras, filename = "xsd_ras.tif", overwrite=TRUE)
```
