# Supplementary material for: Bayesian spatial analysis of factors influencing neonatal mortality and its geographic variation in Ethiopia
Source: PLoS One. 2022 Jul 1;17(7):e0270879. doi: 10.1371/journal.pone.0270879 (PMC9249191; doi:10.1371/journal.pone.0270879)
Supplement: S2 File — (DOCX) [file pone.0270879.s005.docx]

**Model formulation for Bayesian spatial logistic regression**

Let *z(x)* be a random variable observed at location *x,* then a statistical model for *z* is constructed in three components: *z(x)* = *η(x)* + *f (x)* + ϵ*(x).*

The first component, *η(x)*, is the fixed effect of observed covariates with unknown parameters. The third component, ϵ*(x),* represents the measurement error or unstructured error, often ϵ*(x)* ∼ *N(*0*, σ*^2^*)* for unknown parameter *σ* and every location *x*. The second component, *f (x),* is a stochastic process, representing the structured dependence among observations with the assumption that observations closer together in space are more likely to be similar than those further apart. The mathematical formulation to a non-Gaussian response (binary outcome in the current case) can be represented using a link function, *g*, so the response is then modelled with a specified distribution and a mean *g*^−1^*(η(x)* + *f (x))*.

The SPDE is an equation to be solved, and solutions to this equation are stochastic processes whose covariance structure is chosen to satisfy the relationship the SPDE specifies. The SPDE approach involves finding an SPDE whose solutions have the covariance structure and implied precision matrix desired for *f*.

R-INLA provides helper functions to construct the required design and penalty matrices, which is accomplished through the R’s *formula* approach:

formula = Y ~ 1 + X+ f (W; model = spde),

where 1 stands for the intercept term, X is a fixed linear effect, and W represents a smooth spatial effect with the possibility of including additional covariates terms with X as (+ x2+x3...) and noise term with *w* as (+ f(U, model = ’iid’)).

The SPDE model allows the construction of a Delaunay triangulation ([Hjelle and Dæhlen, 2006](#_ENREF_1)) covering the region. These triangulations are partitions of the region into triangles (Supplementary figure 1), satisfying constraints on their size and shape in order to ensure smooth transitions between large and small triangles. Initially, observations are treated as initial vertices for the triangulation, and extra vertices are added heuristically to minimise the number of triangles needed to cover the region subject to the triangulation constraints. These extra vertices are used as prediction locations. This partition is usually called *mesh.* Once the prediction is performed in the selected location, there are additional functions that linearly interpolate the results within each triangle into a finer regular grid. As a result of the process, a faceted surface prediction is obtained, which approximates the true predictive surface. The prediction in INLA is performed simultaneously with the inference, considering the prediction locations as points where the response is missing.

Hjelle, Ø. and M. Dæhlen (2006). Triangulations and applications, Springer Science & Business Media.
