# Supplementary material for: Bayesian spatial analysis of factors influencing neonatal mortality and its geographic variation in Ethiopia
Source: PLoS One. 2022 Jul 1;17(7):e0270879. doi: 10.1371/journal.pone.0270879 (PMC9249191; doi:10.1371/journal.pone.0270879)
Supplement: S3 File — (DOCX) [file pone.0270879.s006.docx]

**Model validation and comparison**

We fit spatial (SPDE) and non-spatial models and compared them using Watanabe-Akaike Information Criterion (WAIC) and Deviance Information Criterion (DIC).

| Model | WAIC | DIC |
| --- | --- | --- |
| Spatial | 1229.11 | 1163.88 |
| Non-spatial | 1234.97 | 1233.26 |

**Model fitness**

Model fitness was assessed for the final Bayesian spatial model using the leave-one-out predictive measures, specifically the conditional predictive ordinates (CPO) and the probability integral transform (PIT) statistics ([Pettit, 1990](#_ENREF_2)).

The conditional predictive ordinates is defined as

CPO*i* = P(Y_i_|Y_(i)_)

where Y = (Y_l_,... ,Yn) is a set of data, Y_(i)_ are the data omitting the i^th^ observation

and P (.|Y_(i)_ is the predictive distribution of a new observation given Y_(i)._ Small values

of CPO indicate that Y_i_ is an outlier in the light of the prior knowledge and fits poorly. Large values indicate a better fit ([Pettit, 1990](#_ENREF_2)).

Probability integral transform is defined as

PIT*i* = P(Y*i*^new^) – (Y*i*/Y-*i*) ([Alene et al., 2022](#_ENREF_1))

PIT measures the probability of a new value Y*i*^new^ being lower than the actual observed value, and larger PIT values indicate a better fit.

There are also internal checks in the inla-program about these assumptions, and

these check will appear as `result$cpo$failure'. In short, if `result$cpo$failure[i] > 0' then some assumption is violated, the higher the value (maximum 1) the more seriously. If `result$cpo$failure[i] == 0, then the assumptions should be ok.

References

Alene, K. A., Elagali, A., Barth, D. D., Rumisha, S. F., Amratia, P., Weiss, D. J., . . . Clements, A. C. A. (2022). Spatial codistribution of HIV, tuberculosis and malaria in Ethiopia. *BMJ Global Health, 7*(2), e007599

Pettit, L. I. (1990). The Conditional Predictive Ordinate for the Normal Distribution. *J. R. Stat. Soc., 52*(1), 175-184 10.1111/j.2517-6161.1990.tb01780.x
